# Associations between Genetic Polymorphisms in *IL-33, IL1R1* and Risk for Inflammatory Bowel Disease

**DOI:** 10.1371/journal.pone.0062144

**Published:** 2013-04-25

**Authors:** Anna Latiano, Orazio Palmieri, Luca Pastorelli, Maurizio Vecchi, Theresa T. Pizarro, Fabrizio Bossa, Giuseppe Merla, Bartolomeo Augello, Tiziana Latiano, Giuseppe Corritore, Alessia Settesoldi, Maria Rosa Valvano, Renata D’Incà, Laura Stronati, Vito Annese, Angelo Andriulli

**Affiliations:** 1 Division of Gastroenterology, IRCCS ‘Casa Sollievo della Sofferenza’, San Giovanni Rotondo, Italy; 2 Gastroenterology and Gastrointestinal Endoscopy Unit, IRCCS Policlinico San Donato, San Donato Milanese, Italy; 3 Department of Pathology, Case Western Reserve University School of Medicine, Cleveland, Ohio, United States of America; 4 Medical Genetic Unit, IRCCS ‘Casa Sollievo della Sofferenza’, San Giovanni Rotondo, Italy; 5 Gastroenterology Unit, AOU Careggi Hospital, Florence, Italy; 6 Department of Surgical and Gastroenterological Sciences, University of Padua, Padua, Italy; 7 Department of Radiobiology and Human Health, Italian National Agency for New Technologies, Energy and Sustainable Economic Development (ENEA), Rome, Italy; 8 Department of Medical and Surgical Sciences, University of Milan, Milan, Italy; INSERM, France

## Abstract

**Background:**

Recent evidence suggests that the IL-33/IL1RL1 axis plays a critical role in several autoimmune and inflammatory disorders; however, its mechanistic role in inflammatory bowel disease (IBD) has not been clearly defined. We investigated the contribution of *IL-33* and *IL1RL1* polymorphisms to IBD risk, and possible correlations with phenotype in an Italian cohort of adult and pediatric patients.

**Methods:**

We evaluated the association of six SNPs in *IL-33* and *IL1RL1* genes, in 805 Crohn’s disease (CD), 816 ulcerative colitis (UC), and 752 controls, using Taqman. IL-33 and IL1RL1 mRNA expression was also analyzed.

**Results:**

Significant allele and genotype associations with *IL-33* rs3939286 were found in CD (*P* = 0.004; *P = *0.035) and UC patients (*P* = 0.002; *P* = 0.038). After stratifying the cohort for age at diagnosis, the differences remained significant only in the IBD adult-onset. Significant associations were also obtained in CD patients with two *IL1RL1* polymorphisms (rs13015714 and rs2058660, *P*<0.015). By combining homo- and heterozygous carriers of the rs13015714 risk allele, differences were still significant for both CD adult- and pediatric-onset. Upon genotype-phenotype evaluation, an increased frequency of extensive colitis in adult UC (*P* = 0.019) and in steroid-responsive pediatric patients (*P* = 0.024) carrying the *IL-33* rs3939286 risk genotype, was observed. mRNA expression of *IL-33* and *IL1RL1* in inflamed IBD biopsy samples was significantly increased.

**Conclusions:**

Common *IL-33* and *IL1RL1* polymorphisms contribute to the risk of IBD in an Italian cohort of adult and pediatric patients, with some influence on sub-phenotypes.

## Introduction

Intestinal mucosal inflammation is a highly regulated process, with a wide repertoire of pro- and anti-inflammatory molecules mediating distinct phases of gut immune responses. Pathologic, chronic intestinal inflammation may arise from an imbalance between different players within this process. As such, the onset and perpetuation of gut inflammation, characterizing inflammatory bowel diseases (IBD), namely Crohn’s disease (CD) and ulcerative colitis (UC), are triggered by constitutive dysregulation of cytokine production [1]. Indeed, a wealth of data indicates the importance of genetic background in regulating the cytokine network in IBD; in fact, polymorphisms of cytokine/cytokine receptor genes have been shown to be associated with the development of IBD [2], [3], [4]. For instance, several studies point out the associations of IBD with genetic polymorphisms involved in the signaling pathway of interleukin (IL)-1 family members, such as IL-1β [5], IL-1 receptor antagonist [6], and IL-18 [7], [8], [9].

IL-33 is a newly characterized cytokine belonging to the IL-1 family with the ability to induce Th2 cytokine production [10] and enhance both Th1 and Th2 [11], as well as Th17 immune responses [12], [13] IL-33′s effects are mediated through the binding of its receptor, IL-1 receptor like 1 (IL1RL1) or T1/ST2, and co-receptor, the IL-1 receptor accessory protein (IL1RAcP), both belonging to the Toll/IL-1 receptor (TIR) superfamily [14]. Several lines of evidence demonstrate the pathogenic role of IL-33 in numerous immune and inflammatory diseases, such as asthma [15], rheumatoid arthritis [12], multiple sclerosis [16] and anaphylaxis [17]. In addition, IL-33 may have dichotomous functions during inflammation, as it has been shown to be protective in a few inflammatory-related conditions, including atherosclerosis [18] and hepatitis [19]. Moreover, genetic dysregulation of the IL-33/IL1RL1 axis appears to be involved in conferring predisposition to several multifactorial diseases, such as Alzheimer’s disease [20], asthma [21], [22], [23], nasal polyposis [24], allergic rhinitis [25], and atopic dermatitis [26].

Recently, several independent researchers have described marked alterations of IL-33 and IL1RL1 expression in IBD [27], [28], [29], [30], [31]. These studies, although focusing on different aspects, consistently demonstrated the upregulation of IL-33 in the inflamed colonic mucosa of IBD patients, with a greater prevalence in UC. Despite the aforementioned evidence suggesting involvement of the IL-33/IL1RL1 axis in the pathologic events leading to the development of IBD, no definitive data regarding the precise role of IL-33 during intestinal inflammation are available.

In the present study we aimed to establish the role of *IL-33* and *IL1RL1* genes in the risk of developing IBD in a well-characterized Italian cohort of adult and early onset IBD patients (805 CD and 816 UC), either by genotyping or by functional studies. Herein, we describe for the first time, the association between the rs3939286 *IL-33* polymorphism with both UC and CD, and between variants of *IL1RL1* with CD. Of note, the aforementioned SNP was also associated with specific clinical UC sub-phenotypes. Taken together, our data further suggest the involvement of IL-33 in the pathogenesis of IBD and provide insight into its possible role in the clinical features of chronic intestinal inflammation.

## Materials and Methods

### Ethics Statement

The study was approved by the Ethical Committee of “Casa Sollievo della Sofferenza” Hospital, San Giovanni Rotondo, and a voluntary written informed consent was obtained from all adult participants and related parents of patients under 19 years of age.

### Patient Recruitment

Demographic and clinical of the Italian IBD patients and controls are shown in **Table 1**. The study group (*n* = 2373) consisted of 805 CD patients, 816 UC patients, and 752 healthy individuals as controls. 303 IBD patients had the initial diagnosis of IBD before their 19^th^ year (155 CD and 148 UC).

**Table 1:** Clinical and demographic characteristics of the study population.

	CD *n* = 805			UC *n* = 816	
***Gender***					
F	321	40%		344	42%
M	484	60%		472	58%
***Duration of follow-up (yr)***					
mean ± SD	8±7			9±7	
median ± SD	6 (1–37)			7 (1–41)	
***Age at diagnosis (yr)***					
mean ± SD	30±14			32±16	
median ± SD	28 (1–80)			30 (1–75)	
Early onset (<19 yrs)	155	20%		148	19%
Adult (≥19 yrs)	622	80%		640	81%
*Missing*	28			28	
≤16 (A1)	130	17%		125	16%
17–40 (A2)	483	62%		456	58%
>40 (A3)	164	21%		207	26%
*Missing*	28			28	
***Disease localization CD, n***					
Ileum (L1± L4)	256	36%			
Colon (L2± L4)	171	24%			
Ileo-colon (L3± L4)	286	40%			
Upper GI (L4)	6	1%			
*Missing*	86				
**Disease extent UC, ** ***n***					
Rectum (E1)				95	12%
Left colon (E2)				374	47%
Pancolitis (E3)				322	41%
*Missing*				25	
***Disease behavior CD, n***					
Inflammatory	475	61%			
Stricturing	209	27%			
Penetrating	94	12%			
*Missing*	27				
**Perianal desease y/n**	143/630	18%		20/765	3%
*Missing*	32			31	
***Extra-intestinal manifestations y/n***	327/446	42%		194/554	26%
*Missing*	32			68	
***Family history of IBD y/n***	55/725	7%		64/729	8%
*Missing*	*25*			*23*	
***Smoking history***					
Yes	229	30%		110	14%
No	402	53%		444	57%
Ex	127	17%		222	29%
*Missing*	*47*			*40*	
***Surgery y/n***	258/527	33%		77/711	10%
*Missing*	*20*			*28*	
***Steroids y/n***	510/295	63%		514/302	63%
*Refractory	73	14%		71	14%
*Responder+dependent	433	86%		438	86%
***IMS (AZT/6MP, CICLO, MTX)***					
Yes	302	38%		217	27%
No	503	62%		599	73%
Non Responder	17	15%		3	23%
Responder	96	85%		10	77%

* Steroids_Resp-DepvsRefrac.

CD: Crohn's disease, UC: ulcerative colitis, IMS: immunosuppressors.

Patients with diagnosis of CD or UC were included in the study based on clinical, endoscopic, and histologic findings according to the Montreal Classification [32]. All individuals were enrolled prospectively from the Gastroenterology Unit at the IRCCS “Casa Sollievo della Sofferenza” Hospital, San Giovanni Rotondo, from centers of the Italian Group of IBD (IG-IBD), and from pediatric centers of the Italian Society of Pediatric Gastroenterology, Hepatology and Nutrition (SIGENP). Clinical data for IBD patients were obtained retrospectively from clinical files. The control group consisted of blood donors and healthy individuals without history of immune-mediated diseases.

### Biopsy Collection

Overall, 29 patients with active IBD, including 15 CD and 14 UC, were used in the present study. Mucosal colonic biopsies were obtained from adult patients undergoing routine colonoscopy at the IRCCS “Casa Sollievo della Sofferenza” Hospital, San Giovanni Rotondo. The activity of disease was evaluated by the Harvey-Bradshaw score (HBI) for CD, and the Mayo score for UC. After obtaining written informed consent, patients with an HBI >4 and a Mayo score >3 were enrolled. Biopsies were taken from inflamed and adjacent non-inflamed regions (10–15 cm distant from pathologic areas). Unaffected areas were defined as mucosal regions without any macroscopic/endoscopic signs of inflammation (*e.g*., discoloration, hemorrhagic appearance, edema, ulceration, or mucinous/fibrinous coating).

### Selection of Tagging Single Nucleotide Polymorphisms (tSNPs) and Genotyping

The selection of SNPs for the present study was made considering previous association studies and the position of the SNPs in the *IL-33* and *IL1RL1* genes. *IL-33* and *IL1RL1* tSNPs were selected using genotypic data from the Caucasian (CEU) Phase II study, available from the HapMap project (http://www.hapmap.org) [33]. SNPs were identified using Haploview software version 4.2 [34] based on solid spine of linkage disequilibrium (LD) (r^2^>0.8, haplotype frequency >5%, minor allele frequency >10%).

This process identified three SNPs for the *IL-33* gene: rs3939286, rs7025417 and rs7044343 (**Figure S1**). The rs3939286 SNP was reported to be associated with nasal polyposis [24] and asthma [21]; the rs7025417 was chosen to tag haplotype block 2; the rs7044343 was selected based on its association with a decreased risk of developing Alzheimer’s disease [20] and in high LD (r^2^>0.8) with the tag SNP rs10975514 of the block 3. By contrast, the significant SNP identified in the CD and UC genome-wide meta-analysis [35], [36], [37] was rs10758669, located at 4.97 Mb on chromosome 9p24 encompassing the *JAK2* gene, with a distance of 1.23 Mb from the selected rs3939286 (6.20 Mb) (**Figure S2**). The three selected SNPs for the *IL1RL1* gene on chromosome 2q12 were rs2058660, rs2310173, and rs13015714 (**Figure S3, Figure S4**). The rs2058660 has already been found to be associated with CD [35]; and the rs2310173 was reported to be associated with UC [36]. In addition, the rs13015714 was found to be significantly associated with an increased risk for celiac disease (2q11–12, *IL18RAP*) [38].

Genomic DNA was extracted from whole blood samples by a standard non-enzymatic method, using the QIAamp DNA Blood Maxi Kit (Qiagen GmbH, Hilden, Germany). Samples were genotyped for the SNPs rs3939286, rs7025417, rs7044343, rs2058660, rs2310173, and rs13015714 using 5′exonuclease TaqMan genotyping assays on an ABI Prism 7900 Real-Time polymerase chain reaction (PCR) System, according to manufacturer’s instructions (Applied Biosystems, Foster City, CA). The genotyping and data management were performed at the Research Laboratory of Gastroenterology Unit of the IRCCS ‘Casa Sollievo della Sofferenza’ Hospital, San Giovanni Rotondo, Italy.

### IL-33 and IL1RL1 mRNA Quantification

Total RNA was extracted from biopsies using Trizol (Invitrogen, Paisley, UK) or RNeasy Mini Kit (Qiagen) according to the manufacturer’s instructions. The amount of RNA was measured by NanoDrop ND-1000 spectrophotometer (NanoDrop Technologies Wilmington, Delaware, USA). The integrity and quality of isolated RNA was determined by Agilent Bioanalyzer 2100 (http://www.chem.agilent.com). In order to preserve the transcriptional profile of tissue specimens, the biopsies were rapidly transferred to RNAlater (Qiagen, Valencia, CA, USA) or snap-frozen in liquid nitrogen and stored until RNA extraction.

### Microarray Data Analysis

Total RNA from biopsy specimens (29 IBD patients) was analyzed with the GeneChip Human Gene 1.0 ST Array System (www.affymetrix.com), which interrogates 28,869 well-annotated genes by using an average of 26 probes per gene. Each sample was processed according to the manufacturer’s instructions. The Affymetrix raw data (.CEL file) were generated using the Affymetrix GeneChip Scanner 3000 7G and analyzed using Partek Genomic Suite Software v. 6.6 (Partek Inc., St. Louis, MO). Briefly, raw intensity values were imported by setting up robust multiarray analysis (RMA) background correction, quantile normalization, and log transformation. Principle component analysis (PCA) was performed as it is an excellent method for visualizing high-dimensional data and identifies outlier samples; mixed model ANOVA was performed in order to generate a comprehensive list of differentially-expressed genes by using a cutoff of P<0.05 for significance of gene-level expression.

For the purpose of this study, we selected the microarray data from all performed comparative analyses for the probe sets encoding the *IL-33* and *IL1RL1* genes.

### Statistical Analysis

Statistical analysis was performed using SPSS software version 14.0 (SPSS, Chicago, IL, USA) and Haploview Software version 4.1 (http://www.broad.mit.edu/personal/mpg/haploview). The genotype frequencies for all investigated polymorphisms were tested for consistency with the Hardy-Weinberg equilibrium. Allelic and genotypic associations of SNPs were evaluated by Pearson's χ^2^ test (or Fisher’s when appropriate) followed by odds ratio and 95% CI. P-values of less than 0.05 were considered significant. Linkage disequilibrium between markers, haplotype structures and haplotype association analyses were also performed. Univariate and logistic regression analyses, on forward stepwise selection procedures, were carried out to correlate various clinical parameters with genotypes and to study pairwise interactions between SNPs of different genes using the number of risk alleles as predictor variables.

## Results

### Case–control Study

The allele and genotype frequencies of the six *IL-33/IL1RL1* SNPs (*IL-33*: rs3939286, rs7025417, rs7044343; *IL1RL1*: rs2058660, rs2310173, rs13015714) were in accordance with the predicted Hardy-Weinberg equilibrium in all subgroups (CD, UC, and controls) (*P*>0.05).

The results for the whole study population are shown in **Tables 2** **,** **3**.

**Table 2:** Association of *IL33* and *IL1RL1* genes markers with Crohn’s disease (CD); case-control study.

		Genotypes frequencies						Alleles frequencies					
		Total CD		Adult CD		Early onset CD		Total CD		Adult CD		Early onset CD	
rs number	Case-Controls frequencies	P value	OR (95% CI)	P value	OR (95% CI)	P value	OR (95% CI)	P value	OR (95% CI)	P value	OR (95% CI)	P value	OR (95% CI)
***IL-33 Gene***													
rs3939286	0.26–0.22	**0.035**	1.24 (1.01–1.53)	**0.029**	1.27 (1.02–1.58)	0.843	1.03 (0.72–1.48)	**0.004**	(1.27 1.07–1.50)	**0.006**	1.28 (1.07–1.53)	0.390	1.13 (0.84–1.52)
rs7025417	0.14–0.15	0.706	0.95 (0.74–1.21)	0.667	0.94 (0.73–1.23)	0.764	0.93 (0.62–1.43)	0.474	0.92 (0.74–1.14)	0.503	0.92 (0.7–1.17)	0.463	0.86 (0.59–1.27)
rs7044343	0.40–0.42	0.224	0.87 (0.71–1.08)	0.100	0.82 (0.66–1.04)	0.795	1.05(0.72–1.52)	0.278	0.92 (0.79–1.06)	0.234	0.91 (0.78–1.06)	0.705	0.95 (0.74–1.22)
***IL1RL1 Gene***													
rs13015714	0.25–0.22	**0.008**	1.32 (1.07–1.62)	**0.039**	1.26 (1.01–1.57)	**0.021**	1.51 (1.06–2.16)	**0.015**	1.23 (1.04–1.46)	**0.040**	1.21 (1.00–1.45)	0.081	1.29 (0.96–1.72)
rs2058660	0.25–0.21	**0.015**	1.29 (1.05–1.58)	0.053	1.24 (0.99–1.54)	**0.046**	1.43 (1.00–2.03)	**0.015**	1.23 (1.04–1.46)	**0.034**	1.21 (1.01–1.45)	0.113	1.26 (0.94–1.68)
rs2310173	0.48–0.47	0.794	0.96 (0.75–1.23)	0.822	0.97 (0.74–1.26)	0.701	0.92 (0.60–1.40)	0.810	0.98 (0.85–1.13)	0.771	0.97 (0.84–1.13)	0.693	0.99 (0.77–1.27)

P value for genotype frequencies was obtained testing for differences between homo- and heterozygous carriers of risk allele *vs* non-carriers of risk allele. OR: corresponding odds ratio and 95% confidence intervals (95%CI). Significant P values (<0.05) are depicted in bold.

**Table 3:** Association of *IL-33* and *IL1RL1* genes markers with ulcerative colitis (UC); case-control study.

		Genotipes frequencies						Alleles frequencies					
		Total UC		Adult UC		Early onset UC		Total UC		Adult UC		Early onset UC	
rs number	Case-Controls frequencies	P value	OR (95% CI)	P value	OR (95% CI)	P value	OR (95% CI)	P value	OR (95% CI)	P value	OR (95% CI)	P value	OR (95% CI)
***IL-33 Gene***													
rs3939286	0.27–0.22	**0.038**	1.24 (1.012–1.52)	0.063	1.22 (0.98–1.52)	0.131	1.32 (0.92–1.89)	**0.002**	1.29 (1.09–1.52)	**0.002**	1.30 (1.09–1.56)	0.139	1.24 (0.93–1.67)
rs7025417	0.14–0.15	0.516	0.92(0.72–1.17)	0.304	0.87 (0.67–1.13)	0.580	1.12 (0.74–1.69)	0.410	0.91 (0.73–1.13)	0.260	0.87 (0.69–1.10)	0.724	1.06 (0.74–1.53)
rs7044343	0.40–0.42	0.055	0.81 (0.66–1.00)	0.062	0.81 (0.64–1.01)	0.393	0.85 (0.58–1.23)	0.352	0.93 (0.80–1.07)	0.434	0.94 (080–109)	0.698	0.95 (0.73–1.22)
***IL1RL1 Gene***													
rs13015714	0.22–0.22	0.239	1.13 (0.92–1.39)	0.245	1.14 (0.91–1.42)	0.172	1.28 (0.89–1.85)	0.675	1.03 (0.87–1.23)	0.714	1.03 (0.86–1.24)	0.250	1.19 (0.88–1.60)
rs2058660	0.22–0.21	0.140	1.16 (0.95–1.43)	0.117	1.19 (0.95–1.48)	0.293	1.21 (0.84–1.74)	0.426	1.07 (0.90–1.27)	0.411	1.07 (0.89–1.29)	0.330	1.16 (0.86–1.56)
rs2310173*	0.49–0.47	0.896	0.98 (0.78–1.23)	0.948	0.99 (0.78–1.26)	0.901	1.02 (0.68–1.53)	0.233	1.09 (0.94–1.25)	0.186	1.10 (0.95–1.28)	0.329	1.13 (0.88–1.45)
rs2310173*	(AA+Aa*vs*aa)	**0.033**	0.77 (0.61–0.98)	**0.023**	0.74 (0.58–0.96)	0.125	0.72 (0.48–1.09)	0.233	0.91 (0.79–1.05)	0.186	0.90 (0.77–1.05)	0.329	0.88 (0.68–1.13)

P value for genotype frequencies was obtained testing for differences between homo- and heterozygous carriers of risk allele (Aa+aa) *vs* non-carriers of risk allele (AA). A = major allele; a = minor allele. OR: corresponding odds ratio and 95% confidence intervals (95%CI). Significant P values (<0.05) are depicted in bold.

### Association between *IL-33* Polymorphisms and IBD

#### The rs3939286 variant of the *IL-33* gene is associated with adult CD and UC patients

A significant allele and genotype association of SNP rs3939286 with CD [*P* = 0.004, OR 1.27 (1.07–1.50); *P* = 0.035, OR 1.24 (1.01–1.53), respectively] and with UC [*P* = 0.002, OR 1.29 (1.09–1.52); *P* = 0.038, OR 1.24 (1.01–1.52), respectively] was observed. After stratifying the CD and UC cohorts in different subgroups according to the age at diagnosis (<19 and ≥19 years), the distribution of allele and genotype frequencies of CD patients [*P* = 0.006, OR 1.28 (1.07–1.53); *P* = 0.029, OR 1.27 (1.02–1.58), respectively], and allelic frequencies of UC patients [*P* = 0.002, OR 1.30 (1.09–1.56)], in comparison to that of control subjects, remained significant only for the adult population (**Tables 2** **,** **3** **)**. All other *IL-33* SNPs investigated in both diseases did not show any significant differences in the allele and genotype frequencies in adult- and early-onset IBD.

### Association between *IL1RL1* Polymorphisms and IBD

#### The rs13015714 and rs2058660 variants of the *IL1RL1* gene are associated with CD patients

A significant difference in allele and genotype frequencies of the rs13015714 SNP in CD population [*P* = 0.015, OR 1.23 (1.04–1.46); *P* = 0.008, OR 1.32 (1.07–1.62), respectively], adult-onset patients [*P* = 0.040, OR 1.21 (1.00–1.45; *P* = 0.039, OR 1.26 (1.01–1.57), respectively], and early-onset patients [*P* = 0.021, OR 1.51 (1.06–2.16): genotype frequency], compared with controls, was observed. Analysis of frequency distribution of the rs2058660 variant revealed significant association with CD patients compared to controls [allelic *P* = 0.015, OR 1.23 (1.04–1.46); genotypic *P* = 0.015, OR 1.29 (1.05–1.58)]. The same genotype was also slightly increased in early-onset CD patients [*P* = 0.046, OR 1.43 (1.00–2.03)], with a similar trend, not reaching statistical significance, in adult patients. Similarly, allele frequency was significantly different in CD adult patients [*P* = 3.45×10^−2^, OR 1.21 (1.01–1.45)] (**Table 2**). No association was found between the *IL1RL1* polymorphisms and the risk of UC disease. However, at recessive model of inheritance, a significant decreased frequency of the rs230173 *IL1RL1* risk genotype [*P* = 0.033, OR 0,77 (0.61–0.98)] was found compared with healthy controls. This association remained significant in the adult subgroup [*P* = 0.023, OR 0,74 (0.58–0.96)] (**Table 3**).

### Association between *IL-33* and *IL1RL1* Polymorphisms and Disease Phenotype

Analysis of genotype-phenotype was performed, interrogating, in each patient, the following clinical features: gender, age at diagnosis, IBD family history, smoking habit, severity and disease localization, presence of perianal fistulas, extraintestinal manifestations, previous abdominal surgery, use and response to medical therapy (mesalamine, corticosteroids, immunosuppressive drugs, azathioprine, 6-mercaptopurine, methotrexate, cyclosporine and infliximab). Patients were classified as responder or non-responder on the basis of a review of medical records as previously described [39].

An increased frequency of extensive colitis (E3vsE1) was observed in UC adult patients carrying the *IL-33* rs3939286 risk genotypes (AG+AA) (47% vs. 33%; *P* = 0.019) compared with those carrying the GG wild-type genotype. Moreover, an increased frequency of the risk genotype in steroid-responsive, early-onset UC patients was also demonstrated (44% vs. 12%; *P* = 0.024) (**Table 4**).

**Table 4:** Association between *IL-33* and rs3939286 polymorphism and disease phenotype.

Gene	SNP	Disease	Phenotype		No. of patientsGG	No. of patients AG+AA	P value	OR (95% CI)
IL-33	rs3939286	UC adult	Localization(E3 vs E1)	Extensive UC (E3)	125 (53%)	112 (47%)	0.019	1.86 (1.10–3.14)
				Proctitis (E1)	56 (67%)	27 (33%)		
		UC early onset	Steroids	No	12 (32%)	26 (68%)	0.001	3.47 (1.57–7.64)
				Yes	64 (62%)	40 (38%)		
			Steroids	Refractory	14 (88%)	2 (12%)	0.024	5.32 (1.14–24.83)
				Responder+Dependent	50 (56%)	38 (44%)		

No other significant correlations of *IL-33* and *IL1RL1* genotypes with either clinical features of patients with UC or efficacy of medical therapy were found. In patients with CD, no significant association of either *IL-33* or *IL1RL1* polymorphisms with any clinical characteristics was demonstrated in both adult- and early-onset patient subgroups.

### *IL-33* and *IL1RL1* Haplotype Analysis

No significant associations between either *IL-33* or *IL1RL1* haplotypes and disease risk was observed (**Table S1**).

### *IL-33* and *IL1RL1* Expression

IL-33 and IL1RL1 mRNA levels in colonic IBD biopsy samples revealed significant differences in UC and CD **(Figure S5)**. In CD patients, IL-33 mRNA transcripts were 1.81-fold significantly increased in inflamed versus non-inflamed mucosa (*P* = 0.0033, FDR = 0.05); in contrast, no significant change in IL1RL1 mRNA expression was found. In UC patients, IL-33 and IL1RL1 mRNA levels showed a significant increase (1.69 fold, *P* = 0.0012, FDR = 0.03; 1.40-fold, *P* = 0.0009, FDR = 0.02, respectively) in inflamed compared to non-inflamed areas.

When comparing the allele dosage with mRNA expression profiles, no differences in IL-33 and IL1RL1 mRNA levels were found (data not shown).

## Discussion

Although the precise etiology remains unknown, it is thought that IBD result from a dysregulated and aberrant immune response to intestinal flora in the context of a genetic predisposition. Moreover, an imbalance of pro- and anti-inflammatory mediators is a critical factor in IBD pathogenesis [40]. Cytokines are key regulators of the intestinal immune system and are known to participate in the disruption of the so-called normal state of controlled inflammation. Early, innate-type cytokines, primarily secreted by the intestinal epithelium as well as activated antigen presenting cells, including dendritic cells and macrophages, actively regulate the inflammatory response in UC and CD. These innate-type cytokines include members of the IL-1 family and have the ability to trigger and differentiate T cells, activating downstream adaptive immune responses. T cell dysregulation in IBD is characterized by aberrant clearance of overreactive and autoreactive cells and an imbalance of Treg/Th1, Th2 and Th17 cell populations. The lack of appropriate regulation from T cells, and/or an over-reactive response from effector T cells, contributes to the development and exacerbation of IBD [41].

Interleukin-33, a novel member of the IL-1 cytokine family [10], has been shown to elicit a Th2- like cytokine response in immunocompetent cells through binding and activation of the interleukin 1 receptor-like 1 [42]. The IL-33/IL1RL1 pathway plays an important role in host defense and in autoimmune, allergic, and chronic inflammatory disorders, such as asthma, dermatitis, rhinitis, arthritis, diabetes mellitus, atherosclerosis, and Alzheimer’s disease [43]. Most recently, IL-33 has been associated with allergic airway inflammation and arthritis in experimental animal models [13], [44]. Interestingly, blocking IL1RL1 receptor signaling has been shown to prevent arthritis development and airway inflammation [45], [15].

The IL-33/IL1RL1 signaling axis has been implicated in IBD in several studies reporting IL1RL1 and IL-33 protein and mRNA expression in IBD patients [28]. An up-regulation of IL-33 in human biopsy specimens, particularly from active UC patients compared to controls was observed [27], [28], [29], identifying epithelial cells, myofibroblasts and macrophages as primary sources of IL-33 within the inflamed tissue of IBD patients. Similarly, an increase in IL1RL1 levels in the gut, mainly associated with the active state of UC, has been described, together with elevated circulating levels of IL1RL1 and IL-33 in IBD patients [28], [29]; *Pastorelli et al*. also showed that anti-TNF therapy modulated IL1RL1 and IL-33 serum levels, increasing the soluble isoform of IL1RL1, making more decoy receptors available, and reducing circulating IL-33 [29].

The research efforts of the IBD International Genetic Consortium, by means of hypothesis-free genome wide association studies (GWAs) [35], [36], [46] and Immunochip project [47], have led to the identification of 163 genomic loci. The majority of loci (67%) confer susceptibility to both Crohn’s disease and ulcerative colitis, with only 30 loci thought to be specific for Crohn’s disease and 23 specific for ulcerative colitis. For almost all of these new loci, the exact gene and/or causal variants remain to be determined. However, GWAs do not have perfect coverage, as about 30% of the common variants are not included on GWAs arrays, leaving gaps that need to be filled by targeted association studies. Moreover, the genetic susceptibility risk explained by GWAs loci to date, is not much higher than 20%, compared with an estimated overall genetic risk of about 40% (based on previous concordance studies in twins) [34], [48]. Of particular relevance to the present study, IBD GWAs have identified genes related to innate immunity as a critical component in the pathogenesis of IBD [49]–[55]. In fact, several recent studies have attempted to document the genetic overlap between IBD and various auto-immune diseases, with approximately 51 IBD genes identified overlapping 23 different diseases, such as multiple sclerosis, rheumatoid arthritis, ankylosing spondylitis, psoriasis, asthma, SLE, and celiac disease [56], yielding significant insight into disease pathogenesis.

Thus, identification of the role of *IL-33* and *IL1RL1* in IBD susceptibility is in line with the recent concept of shared genetic determinants for clinically distinct disorders [57], and notwithstanding the number of IBD susceptibility genes that has increased dramatically over the last several years, genes associated with the IL-33 signaling pathway have not been reported to date. In the International IBD Genetics Consortium meta-analysis for CD and UC, no associated SNPs in *IL-33* were reported, while the rs10758669 variant (position 4971602) of *JAK2* was identified as a CD and UC risk locus (CD_meta_ *P* = 1.00×10^−13^, UC_meta_ *P* = 8.52×10^−13^) [36]. Our selected SNPs in *IL-33* are not in LD with the reported rs10758669 variant (**Figure S2**). Although the selected SNPs have not been reported in the meta-analysis, the significant association with the rs3939286 variant and both CD and UC, particularly in the adult population (*P* = 0.006 and *P* = 0.002, respectively), implicates *IL-33* as a novel IBD susceptibility gene. Interestingly, this genetic association was also found with the UC phenotype. Adult UC patient carriers of the rs3939286 variant were at a higher risk of developing extensive colitis compared to homozygous wild-type carriers (OR = 1.86). Moreover, early-onset patient carriers of the *IL33* rs3939286 risk genotype were significantly more responsive to steroids (44%) compared with noncarriers (12%) (OR = 5.32).

Furthermore, we were able to confirm high levels of IL-33 expressed in the inflamed mucosa of both UC and CD patients, although the risk alleles did not influence the mRNA transcript levels. These data confirm that IL-33 mRNA expression is increased in UC and CD locally, within the colonic mucosa, and are indication of active inflammation.

At present, despite the wealth of data indicating the prominent alterations of IL-33/IL1RL1 expression in the inflamed mucosa of IBD patients, no definitive data have demonstrated the precise role of this axis during gut inflammatory conditions. Interestingly, experimental data obtained using different animal models of intestinal inflammation have produced conflicting results. In fact, IL-33 was shown to potently increase the production of pro-inflammatory cytokines, such as IL-5, IL-6 and IL-17, in SAMP1/YitFc mice, a mixed Th1/Th2 spontaneous model of chronic enteritis [30], whereas exogenous administration of IL-33 showed a protective effect in trinitrobenzene sulfonic acid (TNBS)-induced colitis, a chemically-induced model of colonic inflammation, mostly driven by a Th1 immune response [58]. In addition, three different groups demonstrated dichotomous functions of IL-33 in the setting of acute dextran sodium sulphate (DSS)-induced colitis [59]–[61]. Following epithelial barrier disruption caused by DSS administration, IL-33 appeared to worsen colitis, inducing the recruitment of neutrophils to the site of inflammation [59]–[61], whereas, during the recovery phase, IL-33 showed a prominent effect in promoting mucosal healing [59], [61] and inducing goblet cell proliferation [60], eventually restoring epithelial barrier function.

The dichotomous role of the IL-33/IL1RL1 axis within the context of chronic intestinal inflammation has been previously proposed [62] and may explain the results obtained within the present study regarding the association of the rs3939286 polymorphism of *IL-33* with IBD and with specific UC phenotypes. The rs3939286 polymorphism has been previously shown to be associated with the development of Th2-driven pathologic conditions, such as nasal polyposis, asthma and systemic eosinophilia [21], [22]. As such, it is likely that presence of the rs3939286 polymorphism confers greater bioactivity to IL-33, which is consistent with the exaggerated inflammatory response that characterizes IBD. Moreover, during the onset of UC, a disease mainly dominated by Th2 cytokines, the presence of the rs3939286 variant further promotes a pathogenic Th2 response and may push the phenotype towards a more extensive disease, such as pancolitis. On the other hand, during short term anti-inflammatory treatment, as in the case of steroid treatment, the production of pro-inflammatory cytokines is dampened; in this setting, the increased bioactivity of IL-33 may enhance the healing of mucosal damage, improving the restoration of epithelial barrier integrity, and favor the achievement of disease remission. Again, this may be consistent with the association of the rs3939286 polymorphism to a steroid responsive phenotype, as indicated by our results.

IL-33 exerts its biologic effects through binding of its receptor, the formerly orphaned receptor IL1RL1, also called T1 or ST2 [10]. Two different splice variants of ST2 have been described [63], leading to the synthesis of two different proteins: ST2L, a transmembrane receptor that activates downstream signaling upon IL-33 recognition, and sST2, a soluble molecule that likely serves as a decoy receptor, ultimately blocking IL-33’s biologic effects. The *IL1RL1* gene is located on chromosome 2q12 and a number of IL1 family members, namely IL1R2, IL1R1, IL1RL2, IL18 receptor 1 (IL18R1), and IL18 receptor accessory protein (IL18RAP) reside in the immediate proximity of the *IL1RL1* gene. Interestingly, the *IL18RAP* gene was also recently shown to be associated with celiac disease, a chronic inflammatory disease of the small intestine with autoimmune features [38], [64]. The region spans about 300 kb and is in high linkage disequilibrium. There is evidence for the involvement of genes surrounding *IL1RL1* in human and experimental disease, and therefore the causal locus responsible for genetic association signals from this region is difficult to determine. In fact, it is unclear at present whether the CD-associated (rs2058660) and UC-associated (rs2310173) SNPs in these regions tag two separate loci or one locus.

Our results demonstrate a significant difference in genotype frequencies of the rs2058660 (P = 0.015) and rs13015714 (*P* = 0.008) SNPs in the *IL1RL1* gene between CD patients and controls. The differences were still significant for the rs13015714 risk allele for both adult- (*P* = 0.039) and early-onset disease (*P* = 0.021). In addition, no significant changes in IL1RL1 mRNA expression levels were found in CD biopsy samples; conversely, a significant increase in IL1L1 mRNA transcripts from UC inflamed biopsies was observed (1.40-fold, *P* = 0.0009, FDR = 0.02), without influence of the allele dosage and the expression profiles.

Present literature does not provide robust hints about the functional significance of *IL1RL1* gene polymorphisms studied in the present paper (rs2058660, rs2310173, rs13015714); thus, it is not possible to speculate whether they lead to altered bioactivity of IL1RL1 isoforms. However, our results, showing an association of rs13015714 to CD, classically considered a Th1-driven disease, but not UC, may suggest that the presence of this specific polymorphism may impair the function of the IL-33/IL1RL1 axis. As a consequence, in the presence of a predisposing genetic background to IBD, the rs13015714 polymorphism may polarize the intestinal inflammation towards a Th1-dominated CD phenotype, rather than a Th2-dominated UC phenotype. Interestingly, it has been recently demonstrated that, while IL-33 promotes Th2 cytokine production through IL1RL1 binding, it is also capable of inducing non-polarized immune responses in an IL1RL1-independent manner, implicating the existence of a second IL-33 receptor or, more likely, the presence of IL-33′s direct effect on DNA-transcription [65] through the DNA-binding domain possessed by this cytokine [66]. Therefore, it may be hypothesized that the rs13015714 polymorphism impairs the expression, or IL-33 binding capacity, of both IL1RL1 isoforms. As a consequence, IL-33 may be “free” from its decoy receptor and capable to exert its pro-inflammatory effects, eliciting a IL1RL1-independent inflammatory program and leading to susceptibility of a polarized Th1/Th17-mediated inflammatory condition, such as that observed in CD. In fact, recent evidence suggests that IL-33 has the ability to polarize immune responses towards a Th17 profile in experimental models of arthritis [12], [13].

Indeed, functional data regarding the effects of IL-33′s rs3939286 and IL1RL1’s rs13015714 polymorphisms on respective protein expression and bioactivity are needed to confirm these assumptions, solely based on disease and phenotype associations.

Taken together, our data suggest that risk variants in the IL-33/IL1RL1 system may influence IBD disease susceptibility, particularly in adult populations, and further support the importance of the IL-1 family of cytokines in the predisposition to both CD and UC. Moreover, a specific imbalance between IL-33 and IL1RL1 may play a pathogenic role in this process. The associations between the *IL-33* rs3939286 polymorphism and IBD, and between the *IL1RL1* rs13015714 and CD, further support the overlap in susceptibility loci/genes between IBD and other immune-mediated diseases. Of note, the aforementioned SNPs were reported to be associated with nasal polyposis [24], asthma [21] (rs3939286), and celiac disease [38] (rs13015714). Further investigation as well as targeted functional studies are needed to understand how IL-33 and IL1RL1 variants contribute to disease susceptibility in IBD, and whether presence of these polymorphic markers might have clinical therapeutic implications.

## Supporting Information

Figure S1 **View of the genomic region containing** ***IL-33*** **gene with the selected three single nucleotide polymorphisms.**
(DOC)

Figure S2 **Linkage disequilibrium plot of** ***IL-33*** **gene with genotyped polymorphisms (SNPs) and r^2^ SNP map.** The underlined SNP was selected from the International IBD Genetics Consortium meta-analysis [35].
(DOC)

Figure S3 **Linkage disequilibrium in** ***IL1RL1*** **and surrounding genes on chromosome 2q12 with the selected two single nucleotide polymorphisms.**
(DOC)

Figure S4 **Block 1 and 2 of** ***IL1RL1*** **gene with the selected single nucleotide polymorphism.**
(DOC)

Figure S5 **Expression of** ***IL-33*** **and** ***IL1RL1*** **mRNA in intestinal biopsy samples from ulcerative colitis (UC) and Crohn’s disease (CD) patients.** mRNA levels were calculated as fold increased over respective adjacent noninflamed area (controls). The controls group fold change has value 1.
(DOC)

Table S1 Haplotype frequencies in Crohn’s disease (CD) and ulcerative colitis (UC) patients compared with controls.
(DOC)
