# Supplementary material for: Associations between Genetic Polymorphisms in IL-33, IL1R1 and Risk for Inflammatory Bowel Disease
Source: PLoS One. 2013 Apr 25;8(4):e62144. doi: 10.1371/journal.pone.0062144 (PMC3636262; doi:10.1371/journal.pone.0062144)
Supplement: Figure S1 — View of the genomic region containing IL-33 gene with the selected three single nucleotide polymorphisms. (DOC) [file pone.0062144.s001.doc]

**Figure S1.** View of the genomic region containing *IL-33* gene with the selected three single nucleotide polymorphisms

§

**Locus 9p24.1**


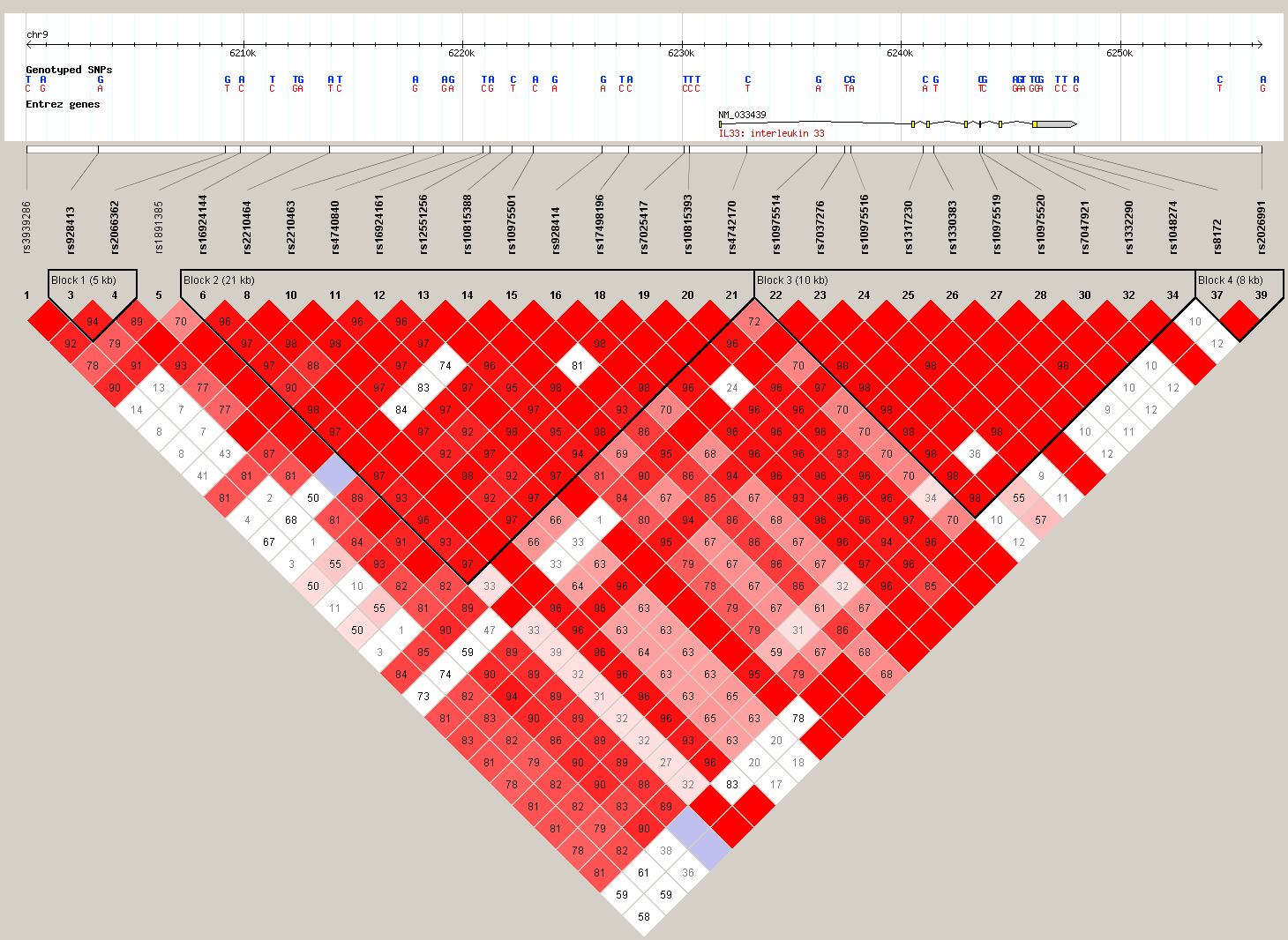


**rs3939286: - asthma**

**- nasal polyposis**

**rs7044343: - associated to Alzheimer**

**- LD with tag SNP rs10975514**

**rs7025417: tag SNP**
