# Supplementary material for: Associations between Genetic Polymorphisms in IL-33, IL1R1 and Risk for Inflammatory Bowel Disease
Source: PLoS One. 2013 Apr 25;8(4):e62144. doi: 10.1371/journal.pone.0062144 (PMC3636262; doi:10.1371/journal.pone.0062144)

**Figure S2.** Linkage disequilibrium plot of *IL-33* gene with genotyped polymorphisms (SNPs) and r^2^ SNP map. The underlined SNP was selected from the International IBD Genetics Consortium meta-analysis (35)


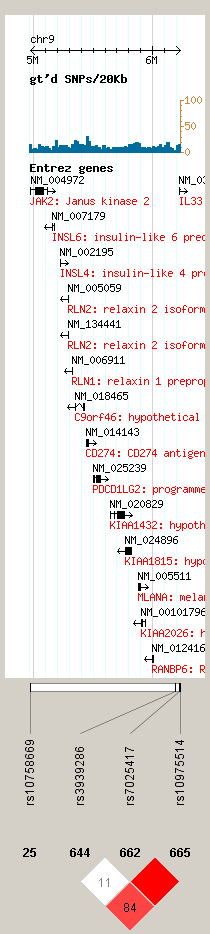

Supplement: Figure S2 — Linkage disequilibrium plot of IL-33 gene with genotyped polymorphisms (SNPs) and r2 SNP map. The underlined SNP was selected from the International IBD Genetics Consortium meta-analysis [35]. (DOC) [file pone.0062144.s002.doc]
