# Supplementary material for: Associations between Genetic Polymorphisms in IL-33, IL1R1 and Risk for Inflammatory Bowel Disease
Source: PLoS One. 2013 Apr 25;8(4):e62144. doi: 10.1371/journal.pone.0062144 (PMC3636262; doi:10.1371/journal.pone.0062144)
Supplement: Figure S3 — Linkage disequilibrium in IL1RL1 and surrounding genes on chromosome 2q12 with the selected two single nucleotide polymorphisms. (DOC) [file pone.0062144.s003.doc]

**Figure S3.**

Linkage disequilibrium in *IL1RL1* and surrounding genes on chromosome 2q12 with the selected two single nucleotide polymorphisms


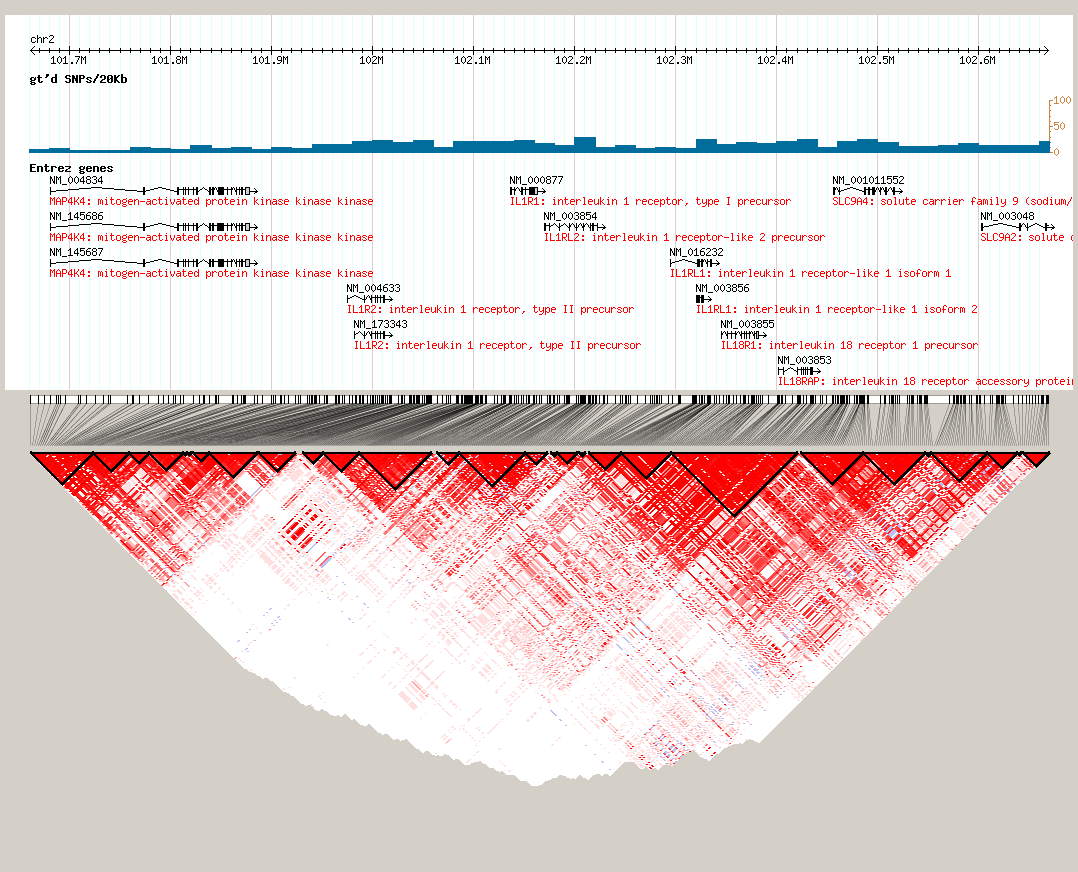
**Locus 2q12**

**rs2310173: meta-analysis UC**

**rs2558660: meta-analysis CD**
