# Supplementary material for: Associations between Genetic Polymorphisms in IL-33, IL1R1 and Risk for Inflammatory Bowel Disease
Source: PLoS One. 2013 Apr 25;8(4):e62144. doi: 10.1371/journal.pone.0062144 (PMC3636262; doi:10.1371/journal.pone.0062144)
Supplement: Figure S4 — Block 1 and 2 of IL1RL1 gene with the selected single nucleotide polymorphism. (DOC) [file pone.0062144.s004.doc]

**Figure S4.**

Block 1 and 2 of *IL1RL1* gene with the selected single nucleotide polymorphism

**
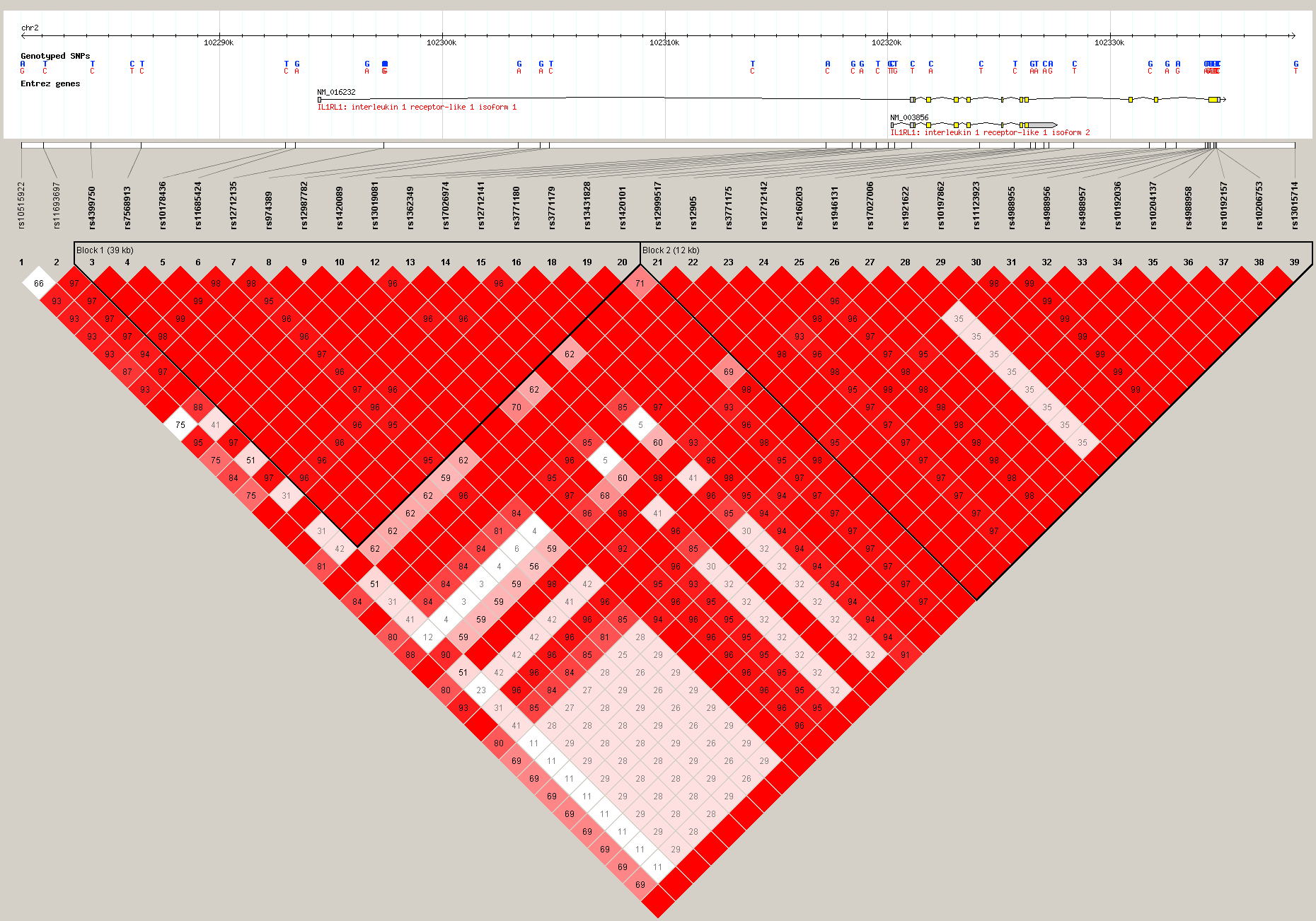
**

**rs13015714: associated to celiac disease**
