# Supplementary material for: Associations between Genetic Polymorphisms in IL-33, IL1R1 and Risk for Inflammatory Bowel Disease
Source: PLoS One. 2013 Apr 25;8(4):e62144. doi: 10.1371/journal.pone.0062144 (PMC3636262; doi:10.1371/journal.pone.0062144)

**Figure S5.** Expression of *IL-33* and *IL1RL1* mRNA in intestinal biopsy samples from ulcerative colitis (UC) and Crohn’s disease (CD) patients. mRNA levels were calculated as fold increased over respective adjacent noninflamed area (controls). The controls group fold change has value 1.

**P* = 0.0012; ***P* = 0.0033; ****P* = 0.0009

CD: Crohn's disease, UC: ulcerative colitis


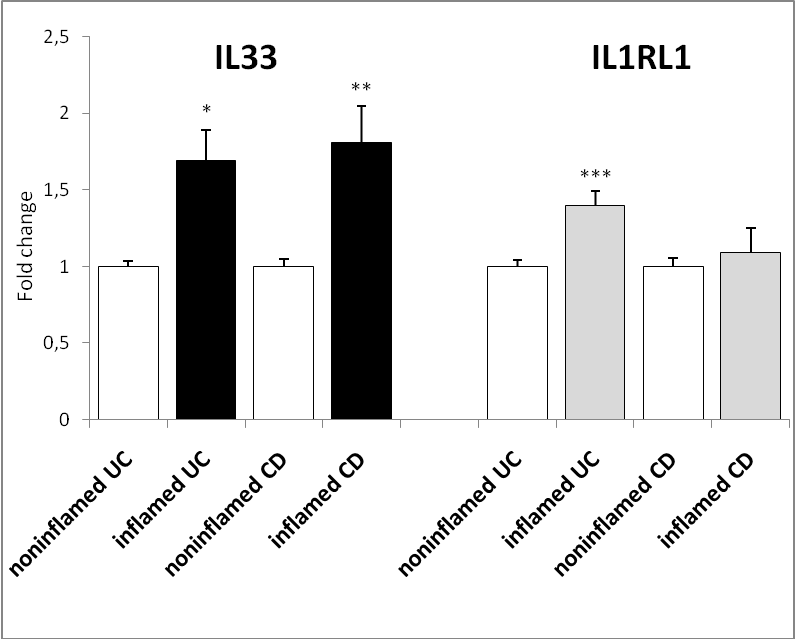

Supplement: Figure S5 — Expression of IL-33 and IL1RL1 mRNA in intestinal biopsy samples from ulcerative colitis (UC) and Crohn’s disease (CD) patients. mRNA levels were calculated as fold increased over respective adjacent noninflamed area (controls). The controls group fold change has value 1. (DOC) [file pone.0062144.s005.doc]
