# Supplementary material for: Associations between Genetic Polymorphisms in IL-33, IL1R1 and Risk for Inflammatory Bowel Disease
Source: PLoS One. 2013 Apr 25;8(4):e62144. doi: 10.1371/journal.pone.0062144 (PMC3636262; doi:10.1371/journal.pone.0062144)
Supplement: Table S1 — Haplotype frequencies in Crohn’s disease (CD) and ulcerative colitis (UC) patients compared with controls. (DOC) [file pone.0062144.s006.doc]

**Table S1. Haplotype frequencies in Crohn’s disease (CD) and ulcerative colitis (UC) patients compared with controls**

|  | | **CD** | | |  | **UC** | |  |
| --- | --- | --- | --- | --- | --- | --- | --- | --- |
| **Haplotypes*** | | **cases/controls**  **frequencies (%)** | | ***P* value** |  | **cases/controls**  **frequencies (%)** | ***P* value** |  |
| ***IL-33*** | |  | |  |  |  |  |  |
| *GTC* | | 36.4/38.1 | | 0.320 |  | 37.5/38.5 | 0.584 |  |
| *GTT* | | 27.6/28.5 | | 0.589 |  | 27.4/28.5 | 0.467 |  |
| *ATT* | | 18.5/16.0 | | 0.068 |  | 18.6/16.1 | 0.061 |  |
| *GCT* | | 9.5/10.5 | | 0.382 |  | 8.4/10.1 | 0.104 |  |
| ***IL1RL1*** | |  | |  |  |  |  |  |
| *GTT* | | 39.6/41.7 | | 0.223 |  | 40.4/42.0 | 0.383 |  |
| *TTT* | | 34.5/35.4 | | 0.581 |  | 36.3/35.2 | 0.534 |  |
| *TGC* | | 12.5/11.0 | | 0.212 |  | 11.8/11.2 | 0.611 |  |
| *GGC* | | 11.8/9.8 | | 0.065 |  | 9.5/9.5 | 0.974 |  |
|  |  | |  | | | | | |

* Data obtained by combining three selected SNPs for *IL-33* gene (rs3939286, rs7025417, rs7044343) and three SNPs for *IL1RL1* (SNPs rs2310173, rs13015714, rs2058660). Only haplotypes with frequency higher than 5% are given.
